# Labelling of Fluids in the Sterile Field During Orthopaedic Surgery: A Quality Improvement Initiative

**DOI:** 10.7759/cureus.72250

**Published:** 2024-10-24

**Authors:** Benedict Sweetman, Zubair Younis, Sarfraz Khan, Jebran Amin, Ghulam Dastagir Faisal Mohammed, Ellen Ellysia Jones, Charlotte Lemaigre, Satya Pydah

**Affiliations:** 1 Trauma and Orthopaedics, Ysbyty Gwynedd Hospital, Bangor, GBR; 2 Orthopaedics, Royal Shrewsbury Hospital, Shrewsbury, GBR; 3 Neurological Surgery, Salford Royal NHS Foundation Trust, Manchester, GBR

**Keywords:** fluid labelling, intraoperative safety, orthopaedic surgery, quality improvement, sterile environment

## Abstract

Background

Intraoperative safety protocols, including proper labelling of syringes, are critical to patient safety in surgical settings. While the Royal Pharmaceutical Society and the Royal College of Anaesthetists provide clear guidelines to prevent medication errors, ensuring consistent compliance with labelling protocols in the fast-paced and complex environment of orthopaedic surgery can still present practical challenges. The absence of proper labelling, combined with the use of multiple fluids such as normal saline, disinfectants, and local anaesthetics, increases the risk of adverse outcomes due to fluid misidentification. This quality improvement project aimed to assess current labelling practices in a district general hospital, identify barriers to compliance, and develop a cost-effective solution.

Methodology

The project was conducted in three orthopaedic theatres over two audit cycles. During the first audit cycle, 30 procedures were observed to assess compliance with labelling guidelines. Compliance was defined as the labelling of all syringes containing fluids present in the sterile field. Following this, an intervention was introduced, using surgical marker pens and sterile stickers for fluid labelling, along with a mandatory “tactical pause and check” and an awareness campaign. Two months later, a second audit of 34 procedures was conducted to evaluate the effectiveness of the intervention.

Results

In the first audit cycle, only three out of 30 procedures (10%) were compliant with labelling guidelines. Following the intervention, compliance increased dramatically to 32 out of 34 procedures (94%). The results were statistically significant (p < 0.05) as determined by Fisher’s exact test. The use of sterile stickers and marker pens proved to be a simple and cost-effective solution that did not interfere with the sterile environment or increase costs.

Conclusions

This study demonstrates that a low-cost intervention using sterile stickers and surgical marker pens can significantly improve compliance with fluid labelling guidelines in orthopaedic surgery, thereby enhancing patient safety. While the intervention was successful, future research should explore more sustainable solutions, such as pre-printed sterile labels, and evaluate the long-term impact of such interventions across various surgical settings. Continuous education and regular audits will be essential in maintaining high compliance rates.

## Introduction

Intraoperative safety protocols are essential for maintaining high standards of care in surgical settings. One area of increasing concern is the proper labelling of syringes containing fluids used during surgery. According to guidelines from the Royal Pharmaceutical Society and the Royal College of Anaesthetists, all syringes must be clearly labelled to prevent medication errors, ensure accurate dosages, and enhance patient safety [1,2]. Several fluids are used by surgeons during orthopaedic surgery. While maintaining sterility is a fundamental skill for all members of the surgical team, labelling syringes in a sterile environment can pose logistical challenges that require careful attention to detail.

The use of intraoperative fluids is integral to many orthopaedic surgeries; normal saline, disinfectant solutions, local anaesthetic cocktails, injectable non-steroidal anti-inflammatory drugs, distilled water, etc. are all used often in combination for a single patient. Despite the clear guidelines, compliance is often difficult when scrubbed due to the need for sterile labels, which are costly and not always readily available.

The absence of proper labelling introduces significant risks, as team members may inadvertently administer the wrong fluid or medication, leading to potentially serious patient outcomes. Proactive prevention of medication errors in perioperative settings is vital to positive patient outcomes [3]. To support this effort, the Association of periOperative Registered Nurses (AORN) has developed a guidance statement to assist clinicians in creating and implementing policies and procedures for safe medication practices in environments where invasive procedures are conducted [4]. Despite these recommendations and practice guidelines, there continue to be nationwide problems with medication labelling compliance [3].

Findings from the 2004 Institute of Safe Medical Practices Medication Safety Self-Assessment, which surveyed over 1,600 hospitals, revealed that less than half (41%) of staff consistently labelled containers (e.g., syringes, basins, and other medication or solution storage containers) in the sterile field [5]. Furthermore, 42% applied labels inconsistently, and 18% did not label medications or solutions in the sterile field at all [5].

Medication-related incidents account for approximately 10% of total reported patient safety incidents in the United Kingdom [6]. An Australian study found that syringe and drug preparation errors contributed to 50.4% of drug error incidents in the operating room, with 18.9% of cases involving correctly labelled drugs being given in error and 20.8% related to mistakes in ampoule selection or labelling [7]. Tragic cases have been reported when medications or solutions were transferred to unlabelled containers, rendering them unidentifiable and causing unsafe practices [8-10].

This quality improvement project aimed to evaluate current labelling practices in the sterile field in orthopaedic theatres in a district general hospital in the United Kingdom, identify barriers to compliance, and introduce a cost-effective, practical solution to improve these practices while maintaining the sterile environment essential for surgery.

## Materials and methods

This quality improvement project was conducted in the orthopaedic department of our busy district general hospital. We have three orthopaedic theatres running every day, two elective and one trauma. Several fluids are used intraoperatively by surgeons. These are loaded into syringes in the sterile field and the practice of labelling them was not standardised. The study was divided into two audit cycles to assess and improve compliance with labelling practices of intraoperative fluids in the sterile field. Both cycles aimed to highlight discrepancies in current practices and promote adherence to recommended guidelines for patient safety.

The data for the first audit cycle were collected over three consecutive working days (April 1, 2024 to April 3, 2024). During this period, 30 orthopaedic surgical procedures were observed, and all of them required the use of intraoperative fluids across three theatres. The cases represented a variety of orthopaedic interventions to ensure a comprehensive assessment of labelling practices across different subspecialty teams. Data were collected through non-intrusive, real-time observations made by an anonymous observer, who remained scrubbed during the surgeries to ensure direct access to the sterile field.

Each surgical procedure was observed from the time the intraoperative fluids (e.g., irrigation solutions, local anaesthetics, and other injectable drugs) were placed on the sterile scrub table until the conclusion of the operation. The observer focused specifically on whether the syringes, bowls, and other containers holding fluids were labelled.

Compliance was defined as the correct labelling of all fluids present on the scrub table. This included fluids used for irrigation, injection, or any other purpose. According to the guidelines, adequate labelling must include both the name of the fluid and the concentration of the preparation. For compliance, all labels needed to be legible and affixed appropriately to each container (e.g., syringe, basin).

A predefined checklist was used by the observer to record data during each procedure. The checklist ensured a standardised approach and allowed for objective data collection. Key items included whether a label was present, and if the label met all required standards (fluid name and concentration).

The primary outcome measure was the percentage of compliance with labelling guidelines during the first audit cycle. Compliance rates were calculated by dividing the number of correctly labelled syringes or containers by the total number of fluid containers used during each procedure. These rates were then averaged across all procedures to give an overall compliance score percentage for the first cycle.

After the first cycle, several interventions were put in place.

Interventions

To address these challenges, a low-cost, practical solution was developed. The surgical marker pen, already available in theatres, comes paired with sterile stickers that can be easily written on and applied to syringes. This eliminated the need for expensive, pre-printed sterile labels. The use of these sterile stickers allowed for fluid labelling in a sterile environment without compromising safety or increasing costs. The scrub nurse was asked to label the fluid immediately after receiving it from a runner in a sterile manner. Additionally, a mandatory “tactical pause and check” was introduced after the opening of surgical sets, during which the theatre team would ensure that all fluids needed for the procedure were available and correctly labelled. It was agreed that mentioning the date and time was redundant as fluid would be labelled immediately after it is drawn into a syringe and discarded at the end of the procedure.

**Figure 1:**
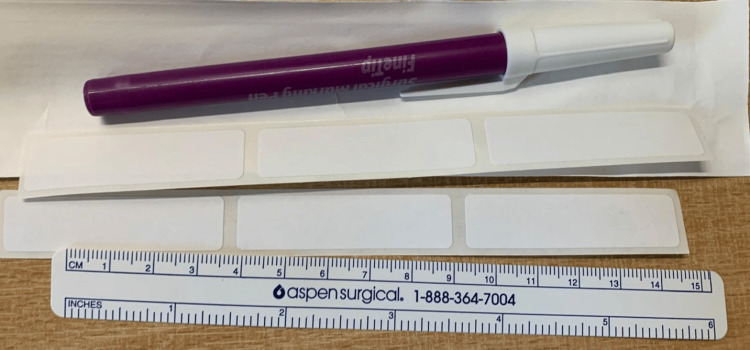
Image from an educational session demonstrating equipment required for labelling: sterile surgical marker, scale, and marking strips. Image credits: Charlotte Lemaigre.

**Figure 2:**
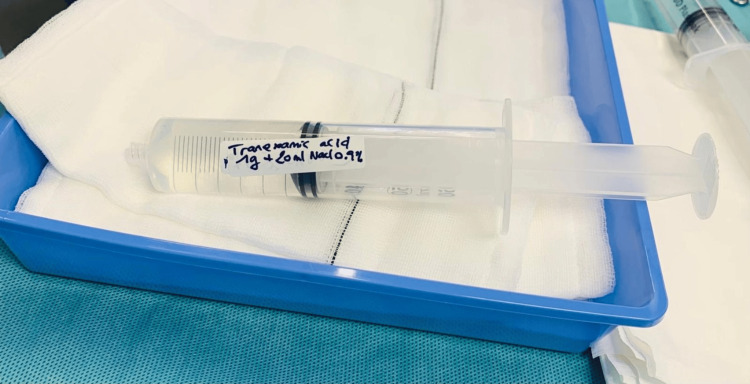
Image from an educational session demonstrating a marked sterile label on a syringe containing tranexamic acid. Image credits: Charlotte Lemaigre.

A multifaceted awareness campaign accompanied this intervention. Posters were displayed in theatres, emails were sent to surgical staff, and educational sessions (Figures 1, 2) were conducted to highlight the importance of fluid labelling.

**Figure 3:**
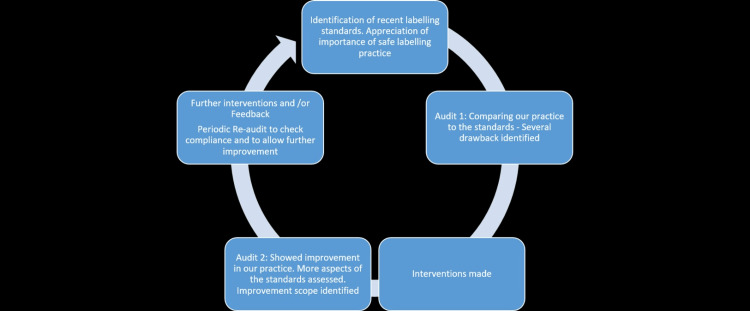
Diagram depicting the audit cycles involved. Image credits: Ghulam Dastagir Faisal Mohammed.

The second cycle was conducted in a similar manner as the first. It was conducted from June 11, 2024, to June 13, 2024. A total of 34 procedures were observed across three theatres and all required intraoperative fluid administration by the surgeon in some form or the other (Figure 3).

No ethical clearance was required for this study, as it was categorised as a clinical audit aimed at improving patient safety within the hospital. All data collected were anonymised, and no patient-specific information was recorded. Additionally, the identity of the operating teams and individual healthcare professionals was not disclosed at any stage of data collection or analysis.

Data were managed on Microsoft Excel (Microsoft Corp., Redmond, WA, USA). Fisher’s exact test was used. P-values <0.05 were considered statistically significant.

## Results

Audit cycle 1

In the first cycle, only three out of 30 procedures (10%) were compliant with labelling requirements. The scrub nurses used paper to write and place syringes or sterile adhesive tape to write and label. The reasons for non-compliance were identified as a lack of available sterile labels, the inconvenience of labelling during critical moments, and human factors affecting the importance of labelling fluid syringes and containers. In addition, labelling information was considered adequate in only one of these three cases.

Audit cycle 2

Two months after the intervention, a second audit was conducted under the same conditions. Over three days, 34 procedures were performed that required fluids. Of these, 32 (94%) were fully compliant with labelling guidelines.

The intervention led to a dramatic increase in compliance with fluid labelling protocols, rising from 10% to 94% in just one month (Tables 1-3).

**Table 1:** Results of both audits showing adequate and inadequate labelling of labelled fluids. Fisher’s exact test statistic value is 0.0695. The result is significant at p < 0.05.

Parameter	Audit 1	Audit 2	P-value
Fluid labelled	3	32	<0.00001
Fluid not labelled	27	2	
Total procedures observed	30	34	

**Table 2:** Results of both audits showing adequate and inadequate labelling of labelled fluids. Fisher’s exact test statistic value is 0.0695. The result is not significant at p < 0.05.

Parameter	Audit 1	Audit 2	P-value
Labelling adequate	1	28	0.0695
Labelling inadequate	2	4	

**Table 3:** Further audit 2 results.

Parameter	Checked by a runner and scrub nurse for expiry and correct fluid	Labelled immediately after drawing up	Checking of labelled fluids during time out	Safely discarded after the procedure
Yes	34	31	34	34
No	0	3	0	0

## Discussion

Medication errors are reported in the literature from time to time. Mistakes have occurred in various settings, such as when a patient under general anaesthesia had his knee injected with epinephrine found in an unlabelled syringe on an operating room preparation table, which was mistaken for bupivacaine, leading to significant complications [10]. Misidentification of a drug because of look-alike drugs, syringe swaps, and confusing, inaccurate, or incomplete drug labels have been found responsible for these errors on many occasions [11]. Up to 86-94% of anaesthesiologists have agreed with the need for standardised drug labels to decrease the incidence of medication errors [12]. While there is considerable literature on perioperative labelling practices, and compliance with these guidelines is generally high [8], there is a notable lack of research focusing on labelling within the sterile field. Despite the emphasis on safety in perioperative labelling, literature on labelling practices in the sterile field is scarce, highlighting the need for further research and standardisation in this critical area.

The AORN has played a leading role in tackling this issue, issuing a revised guidance statement in 2004 that outlines risk reduction strategies and provides sample protocols [4]. AORN also provides (at no charge to members) a Safe Medication Administration Tool Kit containing educational materials, resources, and testing materials [13].

This quality improvement project demonstrates that a simple, low-cost solution can have a profound impact on compliance with safety protocols in the operating theatre. The use of sterile stickers and surgical marker pens allowed for easy and effective labelling of intraoperative fluids without significantly altering the theatre workflow or adding to costs. Studies conducted by Sheridan and Brown-Brumfield and DeLeon have similarly observed an improvement in compliance with these methods, further supporting their effectiveness in enhancing safety and efficiency [3,8].

An essential aspect of this study was the accompanying multifaceted awareness campaign aimed at fostering a culture of safety around fluid labelling. Posters in operating theatres, emails to the surgical team, and educational sessions collectively served as visual reminders, timely updates, and interactive platforms to emphasise the critical importance of accurate labelling and promote best practices for safety [14].

Inadequate labelling in the operating room is often due to time constraints, lack of standardised protocols, human error, and poor communication among surgical staff. Labelling, especially in fast-paced environments, can lead to incomplete or illegible labels, increasing the risk of medication errors. Studies such as those by Sheridan and Brown-Brumfield and DeLeon highlight that standardising labelling practices with pre-printed sterile labels significantly improves compliance and reduces errors [3,8]. Additionally, better communication and regular education on labelling protocols are essential to ensure consistent safety in the operating room.

Labelling was not immediate after drawing up fluids due to time pressures, task prioritisation, and workflow interruptions in the operating room, where staff often focused on preparing medications quickly over labelling. To address this, we introduced a process where the circulating nurse (runner) reminded the scrub team to label fluids immediately before proceeding to the next task. This real-time reminder ensured prompt labelling, reducing the risk of mix-ups and fostering a more consistent and safer workflow.

In this study, we agreed not to include the date and time on the labels, as fluids were drawn just before surgery, double-checked by two people, and discarded immediately after the procedure. This decision was made to save time and avoid redundancy, as the rapid turnover of fluids made this information unnecessary for safety in this specific context.

While sterile stickers and surgical markers provide a practical and low-cost alternative for fluid labelling in the operating room, they come with certain limitations. One issue is the use of permanent ink, which may smear or fade, potentially rendering the label unreadable over time, especially in fluid-rich environments. According to drug labelling guidelines, labels must include essential information such as the drug name, concentration, and expiration time. However, hand-written labels often lack the consistency and clarity of pre-printed sterile labels, which may include colour coding and standardised fonts for readability and safety.

Additionally, the process of manually labelling each syringe during surgery takes time, which can have cost implications in a fast-paced operating theatre setting. With operating theatre costs averaging £20 per minute [15], spending just a few extra minutes on labelling can significantly increase the total expense of a procedure. Pre-printed sterile labels, while initially more expensive, might prove to be more cost-effective over time, reducing the need for manual labour and avoiding potential errors associated with hand-written labels. Some studies have demonstrated more compliance with the pre-printed labels by the scrub personnel [16]. However, a thorough cost-benefit analysis is needed to evaluate whether pre-printed labels are indeed a cheaper and more efficient solution.

This study has several limitations. First, the small sample size, limited to a single hospital, may not fully represent labelling practices in other institutions or specialties. The study was also conducted over a relatively short time frame, which could limit the generalisability of the findings. Additionally, while the intervention led to significant improvement, the sustainability of the changes was not tested beyond the initial follow-up period. Behavioural change in clinical settings often requires long-term reinforcement, and without ongoing education and regular audits, compliance may decrease over time. Lastly, the study did not assess patient outcomes directly, so while labelling compliance improved, further research is needed to establish a clear link between these improvements and reduced medication errors or enhanced patient safety. Regular audits, continuous staff education, and the inclusion of labelling checks in standard operating procedures will be essential in maintaining high levels of compliance.

## Conclusions

This study demonstrates that a simple, cost-effective intervention can significantly improve compliance with intraoperative fluid labelling guidelines, thereby enhancing patient safety in orthopaedic theatres. The use of surgical marker pens and sterile stickers offers a viable alternative to expensive pre-printed sterile labels, particularly in resource-constrained settings.

Future improvements could focus on introducing pre-printed sterile labels as a long-term solution, reducing the burden on theatre staff while maintaining compliance. Further research may explore the application of similar interventions in other surgical specialties and settings. Continuous education, regular audits, and reinforcement of safety protocols will be essential to sustaining the gains achieved through this quality improvement initiative.
